# Local Disease-Free Survival Rate (LSR) Application to Personalize Radiation Therapy Treatments in Breast Cancer Models

**DOI:** 10.3390/jpm10040177

**Published:** 2020-10-17

**Authors:** Gaetano Savoca, Marco Calvaruso, Luigi Minafra, Valentina Bravatà, Francesco Paolo Cammarata, Giuseppina Iacoviello, Boris Abbate, Giovanna Evangelista, Massimiliano Spada, Giusi Irma Forte, Giorgio Russo

**Affiliations:** 1 Institute of Molecular Bioimaging and Physiology (IBFM-CNR), 90015 Cefalù (PA), Italy; savoca.gaetano@gmail.com (G.S.); marco.calvaruso@ibfm.cnr.it (M.C.); valentina.bravata@ibfm.cnr.it (V.B.); francesco.cammarata@ibfm.cnr.it (F.P.C.); giusi.forte@ibfm.cnr.it (G.I.F.); giorgio.russo@ibfm.cnr.it (G.R.); 2 Medical Physics Department, ARNAS-Civico Hospital, 90100 Palermo, Italy; giuseppina.iacoviello@arnascivico.it (G.I.); borisfederico.abbate@arnascivico.it (B.A.); 3 Radiation Oncology, ARNAS-Civico Hospital, 90100 Palermo, Italy; giovanna.evangelista@arnascivico.it; 4 Oncology Unit, Fondazione Istituto G. Giglio, 90015 Cefalù (PA), Italy; massimiliano.spada@hsrgiglio.it

**Keywords:** breast cancer, radiotherapy, LSR, LQ model, personalized treatments

## Abstract

Cancer heterogeneity represents the main issue for defining an effective treatment in clinical practice, and the scientific community is progressively moving towards the development of more personalized therapeutic regimens. Radiotherapy (RT) remains a fundamental therapeutic treatment used for many neoplastic diseases, including breast cancer (BC), where high variability at the clinical and molecular level is known. The aim of this work is to apply the generalized linear quadratic (LQ) model to customize the radiant treatment plan for BC, by extracting some characteristic parameters of intrinsic radiosensitivity that are not generic, but may be exclusive for each cell type. We tested the validity of the generalized LQ model and analyzed the local disease-free survival rate (LSR) for breast RT treatment by using four BC cell cultures (both primary and immortalized), irradiated with clinical X-ray beams. BC cells were chosen on the basis of their receptor profiles, in order to simulate a differential response to RT between triple negative breast and luminal adenocarcinomas. The MCF10A breast epithelial cell line was utilized as a healthy control. We show that an RT plan setup based only on α and β values could be limiting and misleading. Indeed, two other parameters, the doubling time and the clonogens number, are important to finely predict the tumor response to treatment. Our findings could be tested at a preclinical level to confirm their application as a variant of the classical LQ model, to create a more personalized approach for RT planning.

## 1. Introduction

Based on the most updated estimations from the World Health Organization (WHO), almost 10 million people died from cancer in 2018, with 70% of those deaths occurring in countries with low and middle incomes, despite a steady improvement of diagnostic and therapeutic techniques in cancer care [1].

To date, according to the classical concept of “one size fits all”, cancer patients with different histological subtypes have been usually treated on the basis of general therapeutic criteria, without taking into account the huge and complex heterogeneity that can be found within neoplasms belonging to the same class. After the introduction of gene expression profiling and the development of DNA and RNA sequencing techniques, it has become increasingly evident that tumors of the same organ can be divided into subtypes, with specific and singular molecular profiles that make them resistant to those therapeutic approaches that may be instead effective for tumors with similar histological traits. These findings have led to the development of the known “tailored therapies”, including surgery, chemotherapy, immunotherapy, and radiotherapy (RT), with the aim of a personalized medicine that considers specific tumor features for each patient [2].

The principal purpose behind RT is to deliver a specific radiation dose to a defined tumor target, and to strike all cancer cells by using high-energy photons or hadrons, thus avoiding as much as possible adverse effects to the surrounding healthy tissues. Moreover, contrary to chemotherapy, which has evolved with a continuous discovery of new drugs, RT has been remained almost the same since its origins, and advances have mainly affected clinical technologies. These have led to a physical and conformational customization of the treatments, rather than a biologically-based personalization of the total dose to be delivered to patients [3]. Nowadays, different types of neoplasms are treated by means of RT, including breast cancer (BC); however the same total dose is delivered to patients with the same organ tumor, without taking into consideration the histological and molecular differences that make some tumor subtypes more resistant to radiation treatments [4].

Cell radiosensitivity is a parameter of high importance to obtain biological effectiveness following RT; being variable among tissues, it may explain the difference between a responsive target and non-responsive one. Often radiosensitization plays a crucial role as an adjuvant for RT through the use of synthetic or natural chemical compounds, able to increase the radiosensitivity of cancer cells and thus the RT’s effectiveness [5].

Thus, variable tumor radioresistance and cancer heterogeneity represent “two faces of the same coin” and, as happens for chemotherapy, the concept of personalized medicine or targeted therapy should be extended also to RT, in order to find the appropriate treatment planning that best fits specific cancer subtypes. In particular, according to international guidelines, BC is commonly treated with RT exposing patients to a total dose of 50–60 Gy, with a fractionation of 2 Gy/day for 5 days/week. Moreover, in some cases of high recurrence risk, a radiation boost of 10–20 Gy could be delivered in order to optimize the local control on surgical bed [6,7]. However, BC is a heterogeneous group of tumors, and based on their morphological, immunophenotypic, and molecular profiles, it is classified in at least four major subgroups: luminal A, luminal B, HER2-enriched, and basal-like breast cancer (BLBC). BLBCs are similar and are often used to indicate another group of neoplasms, namely triple-negative breast cancers (TNBCs), which are characterized by the lack of expression of the estrogen receptor (ER), progesterone receptor (PR), and the human epidermal growth factor receptor 2 (Her2/neu). However, it is important to underline that this association is somewhat controversial. In fact, BLBCs and TNBCs often display different molecular profiles, where the former overexpress basal markers, such as the cytokeratins 5, 6, and 17, and the latter do not always do so. Thus, the four BC subgroups may be enlarged, but the picture is even more complex, considering that TNBCs can be further divided into at least four distinct cancer subtypes with different genomic profiles and prognoses [8,9,10].

TNBCs account for 10–15% of BC, and they are characterized by a bad prognosis and a high recurrence rate. Patients cannot be treated with endocrine therapy or trastuzumab because of the lack of specific tumor markers, and currently no exclusive strategies are available for triple-negative forms. Therapy is mainly based on neoadjuvant and adjuvant chemotherapy administration, but the gold standard chemotherapy has not been established yet [11,12]. In addition, TNBCs show a certain level of radioresistance, as demonstrated by the rate of relapses that occur in between 25% to around 40% of the cases after RT [13,14,15,16,17]. Nonetheless, as mentioned above, TNBCs are currently treated with the same total dose provided to the other, less aggressive BC subtypes [18,19]. Thus, the increasing evidence of different responses to RT in terms of tumor radioresistance, should lead to rescheduling existing radiation therapy protocols, and take into account the different cancer molecular profiles affecting the RT cellular response [20].

In order to understand the rationale behind the choice to deliver a specific total dose for a determined class of tumors, we should take into account the linear quadratic (LQ) model. The LQ model represents a powerful tool used by medical physicists to describe the in vitro dose response effect of a certain cell line treated with increasing doses of ionizing radiation (IR) [21].

It basically puts into a relationship the cell survival and the dose delivered, following this formula:

$$\frac{S\left(D\right)}{S\left(0\right)}=e^{-\alpha D-\beta D^{2}}$$

where *S(D)* is the survival fraction at the dose *D*, *S*(0) is the survival fraction at the dose *D* = 0, the terms *α* and *β* represent the two forms of damage induced by radiation, and *D* is the dose value measured in Gy. With respect to *α* and *β*, the first refers to the lethal damage caused by a single incident particle. The latter represents the multiple-hit damage that is not fatal per se and could be repaired, caused by different radiation tracks, and its value is proportional to the dose squared. Considering that in clinical practice, the total dose delivered is divided into n fractions for each type of cancer to treat, the previous formula can be adjusted as follows:

$$S=e^{\left(-\alpha d-{\beta d}^{2}\right)n}=e^{-\left(\alpha d+{\beta d}^{2}\right)n}=e^{-D\left(\alpha +\beta d\right)}$$

where d is the single dose per fraction, and to simplify the notation we denote using *S* the ratio *S*(*D*)/*S*(0). Plotting this formula on a log scale, it gives a typical quadratic response curve [22].

An extension of the LQ model is the “generalized LQ model” [23], which starts from the same LQ mathematical basis but takes into consideration other parameters that generalize it. Therefore, a model based on the generalized LQ model and the statistical model of Poisson was developed to calculate the local disease-free survival rate (LSR). In radiotherapy, the LSR is a parameter used to evaluate the disease-free percentage of the patient population, and it refers to local (or loco-regional) relapses after primary cancer treatment. The LSR depends on dose fractionation and on some biological effects, such as repopulation, repair, tumor cell doubling time, and number of tumor clonogens. It also depends on the statistical distribution model used. One model that can take into account the biological effects is the Poisson model. Thus, the LSR is related to the cell clonogenic capacity during IR treatment and to cell survival (SF) exponential behavior with temporal follow-up [24].

The aim of this work is to apply the generalized LQ model to customize the radiant treatment plan for BC, not only considering the geometric and conformational features of the tumor, but also extracting some characteristic parameters of intrinsic radiosensitivity that are not generic but may be exclusive for each cell type. On this purpose, we tested the validity of the generalized LQ model by treating in vitro four breast cancer cell cultures (both primary and immortalized) with a clinical beam used for X-ray RT. Breast cancer cells were chosen on the basis of their estrogen (ER), progesterone (PR), and ERBB2 (HER2) receptor assets, in order to simulate a differential response to RT between triple negative breast and luminal adenocarcinomas. Similarly, the MCF10A breast epithelial cell line was utilized as healthy control.

We show that the RT plan setup, based only on α and β values (hence the α/β ratio), could be limiting and misleading. Indeed, two other parameters—the doubling time (a surrogate of the tumor cell repopulation capability) and the clonogen numbers (on which the repopulation potential relies on)—are important as well as the α/β ratio, in order to finely predict the tumor response to treatment.

Considering the LSR as a function of doubling time (T_d_) and clonogen numbers (K), together with the α/β ratio, our approach could be tested at a preclinical level, to confirm its application as a variant of the classical LQ and create a more personalized approach for radiotherapy planning.

## 2. Materials and Methods

### 2.1. Cell Cultures and Radiation Treatments

The human breast adenocarcinoma MCF7 and MDA-MB-231 cell lines, and the human non-tumorigenic breast epithelial MCF10A cell line, were purchased from the American Type Culture Collection (ATCC, Manassas, VA, United States) and maintained in culture according to standard conditions and procedures, as previously described [25].

The two primary human BC cell lines, named BCpc7 and BCpcEMT, were isolated from breast surgery specimens of infiltrating ductal carcinoma and cultured as previously described [26,27]. The Ethical Committee of Fondazione Istituto G. Giglio, Cefalù-Italy (number of protocol: C.E.2012/16) approved the study and the consent procedure, both of which were performed according to the Helsinki declaration.

Cell irradiations were performed with photon beams (X-rays) of 6 MV nominal energy and doses of 1, 2, 3, and 4 Gy, using a medical linear accelerator (Siemens Medical Systems, Concord, CA, USA). The Linac calibration, irradiation setup, and dose distribution were conducted as previously described [25].

### 2.2. Clonogenic Survival Assay, Dose Response Curves, and Alfa and Beta Parameter Calculations

In order to calculate the surviving fraction (SF) values post-irradiation, clonogenic assays were performed as previously reported [25]. For each BC cell line, the untreated cell sample, seeded and maintained in culture in parallel, was used to calculate the plating efficiency (PE) and the clonogen numbers (*k* value). Data normalization was performed with respect to the untreated sample. In order to carry out dose–response curves, the clonogenic survival data were analyzed using a non-linear regression, with the following multi-parameter equation:

$$SF=S\left(x\right)/S\left(0\right)=e^{-\alpha x-\beta x^{2}}$$

from which we obtained the α[$Gy^{-1}$] and *β*[$Gy^{-2}$] parameters, extracted from the fit with their own standard deviation, and where *S*(0) is the zero-dose surviving fraction, obtained from a previous second-order fit, and as previously described [28].

The uncertainty of SF values was obtained by taking the partial derivative or the method of propagation of errors. In particular,

$$\sigma \left(SF\right)\;=\sqrt{{\left(\frac{\partial SF)}{\partial x}\right)}^{2}\cdot {\left(\Delta x\right)}^{2}+{\left(\frac{\partial SF}{\partial \alpha }\right)}^{2}\cdot {\left(\Delta \alpha \right)}^{2}+{\left(\frac{\partial SF}{\partial \beta }\right)}^{2}\cdot {\left(\Delta \beta \right)}^{2}+{\left(\frac{\partial SF}{\partial S\left(0\right)}\right)}^{2}\cdot {\left(\Delta S\left(0\right)\right)}^{2}}$$

where the individual terms are

$$\left(\frac{\partial SF)}{\partial x}\right)=-S\left(0\right)e^{-\left(\alpha x+\beta x^{2}\right)}\left(\alpha +2\beta x\right)$$

$$\left(\frac{\partial SF)}{\partial \alpha }\right)=-S\left(0\right)xe^{-\left(\alpha x+\beta x^{2}\right)}$$

$$\left(\frac{\partial SF)}{\partial \beta }\right)=-S\left(0\right)x^{2}e^{-\left(\alpha x+\beta x^{2}\right)}$$

$$\left(\frac{\partial SF)}{\partial S\left(0\right)}\right)=e^{-\left(\alpha x+\beta x^{2}\right)}$$

### 2.3. Local Disease-Free Survival Rate (LSR) Model

Post-operative RT is commonly utilized to reduce local relapses of BC. Generally, a dose per fraction of 2.0 Gy is used in 25 fractions for 5–6 weeks of a standard treatment. In recent times, it has been observed that the α/β ratio for BC may be lower than previously calculated [29]. Hypofractionation schemes represent an interesting alternative for early BC irradiation treatments. With respect to a regular fractionation scheme, hypofractionation is characterized by a small number of fractions with higher doses (more than 2 Gy for fraction), thus allowing the maintenance of high levels of LSR in a shorter treatment period. As already mentioned in the previous paragraph, the model used to evaluate the LSR is the generalized LQ model [23,24]. In formulas, it appears as

(1) $$SF=\;e^{-E}$$

(2) $$E=\alpha D+\beta GD^{2}-\gamma T$$

where *S* is the surviving fraction, the *α* and *β* parameters represent the specific radiosensitivity, and *G* is the dose protraction factor or time factor of Lea-Catcheside [30]:

(3) $$G=\left(2/D^{2}\right)\int_{-\infty }^{\;\infty \;}D^{\prime }\left(t\right)dt\int_{-\infty }^{\;t}e^{-\lambda \left(t-t^{\prime }\right)}D^{\prime }\left(t^{\prime }\right)\;{\mathrm{dt}}^{\prime }$$

where *D’*(*t’*) describes the variation in dose-rate throughout the radiotherapy treatment course, and λ takes into account the repair rate of the characteristic damage. In external radiation therapy, the delivery time of radiation dose is generally shorter than the cancer cell repair interval; therefore, the total dose *D* = *n × d* and *G* factor = 1/*n*, where *n* is the number of fractions and *d* is the single dose for each fraction.

The quantity γ is the effective rate of repopulation of cancer cells; $\;\gamma =\ln2/T_{d}$, and $T_{d}$ represents the doubling time of cancer cells, a known and calculable parameter for each cell line. The total duration of treatment (*T*) can be easily calculated, multiplying the number of fractions of the treatment by 1.4 (considering five fractions for week).

Therefore, the LSR for BC is calculated from Equation (1), using the Poisson hypothesis:

(4) $$\mathrm{LSR}(D)=\;e^{-k\cdot SF}$$

where *k* represents the number of tumor clonogens. The LSR dependence on the *k* factor changes has been investigated by other groups [23,31]. Due to the *k* factor heterogeneity, our experimental approach aimed first to compare two LSRs obtained using two different *k* values, which were previously described in literature, namely *k* = 36 [31] and *k* = 14.5 [23]. Moreover, in our work, we propose a method to measure *k* experimentally.

The LSR is calculated from Equations (1), (2), and (4), as:

(5) $$\mathrm{LSR}(D)\;=\;e^{-k\cdot e^{\left(-\alpha D-\beta D^{2}+\gamma T\right)}}$$

The uncertainty in LSR values is obtained by taking the partial derivative or the error propagation method. Therefore, errors in LSR (*D*) will be obtained from the formula

(6) $$\sigma \left(LSR\right)=\sqrt{{\left(\frac{\partial LSR)}{\partial D}\right)}^{2}\cdot {\left(\Delta D\right)}^{2}+{\left(\frac{\partial LSR}{\partial \alpha }\right)}^{2}\cdot {\left(\Delta \alpha \right)}^{2}+{\left(\frac{\partial LSR}{\partial \beta }\right)}^{2}\cdot {\left(\Delta \beta \right)}^{2}+{\left(\frac{\partial LSR}{\partial k}\right)}^{2}\cdot {\left(\Delta k\right)}^{2}}$$

The analysis of the individual terms leads to:

$$\left(\frac{\partial LSR)}{\partial D}\right)=k\left(-\alpha -\frac{2\beta D}{n}\right)\left(-exp\left(-k\cdot e^{\left(-\alpha D-\frac{\beta D^{2}}{n}+\gamma T\right)}-\alpha D-\frac{\beta D^{2}}{n}+\gamma T\right)\right)$$

$$\left(\frac{\partial LSR)}{\partial \alpha }\right)=kD\left(-exp\left(-k\cdot e^{\left(-\alpha D-\frac{\beta D^{2}}{n}+\gamma T\right)}-\alpha x-\frac{\beta D^{2}}{n}+\gamma T\right)\right)$$

$$\left(\frac{\partial LSR)}{\partial \beta }\right)=\frac{{kD}^{2}\left(exp\left(-k\cdot e^{\left(-\alpha D-\frac{\beta D^{2}}{n}+\gamma T\right)}-\alpha D-\frac{\beta D^{2}}{n}+\gamma T\right)\right)}{n}$$

$$\left(\frac{\partial LSR)}{\partial k}\right)=-exp\left(-k\cdot e^{\left(-\alpha D-\frac{\beta D^{2}}{n}+\gamma T\right)}-\alpha D-\frac{\beta D^{2}}{n}+\gamma T\right)$$

### 2.4. Statistical Analysis

For each BC cell type used in this study, three different experiments were performed and the Chi square (**χ**^2^) test was applied, referring to the mean of the surviving fractions obtained in three experiments. When the tolerated error was set to 5% (α = 0.05) and the degrees of freedom were 4, referring to the table of the Chi-squared distribution, we obtained a value equal to 9.49, which is greater than our value of 1.00. Therefore, we concluded that we can accept the null hypothesis or that the data sets follow a Gaussian probability distribution, or more simply, they belong to the same statistical set. Moreover, in our analysis, we used the adjusted *R*^2^ as the main goodness index of the non-linear regression curve (*SF = e*^(*−αx−βx*^2)) for the *SF*(*D*) data, with the known meaning that an *R*^2^ close to 1 means that the data predict the value of the dependent variable in the sample, while if it is equal to 0 it means that they do not. The **χ**^2^ and the adjusted *R*^2^ values for each cell line are shown in the Supplementary Materials, Table S2.

## 3. Results

### 3.1. Radiobiological Characterization of Breast Cancer(BC) Cell Lines and Primary Cultures

Five different BC cell populations were used in this study, three immortalized and two primary cell cultures, in order to broadly represent the great BC heterogeneity. On this basis, cell lines were chosen according to their estrogen (ER), progesterone (PR), and ERBB2 (HER2) receptor expression: the ER^+^/PR^+^/HER2^-^ MCF7 cell line; the ER^-^/PR^-^/HER2^-^, metastatic, and radioresistant MDA-MB-231 cell line [20]; and the ER^-^/PR^-^/HER2^-^, non-tumorigenic MCF10A cell line, chosen as a healthy control [32]. In addition, to closely resemble a real pathological setting, two primary cultures were used: the TNBC ER^-^/PR^-^/HER2^-^ BcPcEMT and the ER^+^/PR^+^HER2^-^ BcPc7, previously isolated and characterized at the phenotypic and molecular levels by our research group. In particular, the BcPcEMT cells were characterized by a strong “epithelial to mesenchymal transition” (EMT) phenotype [25,26,33].

**Figure 1:**
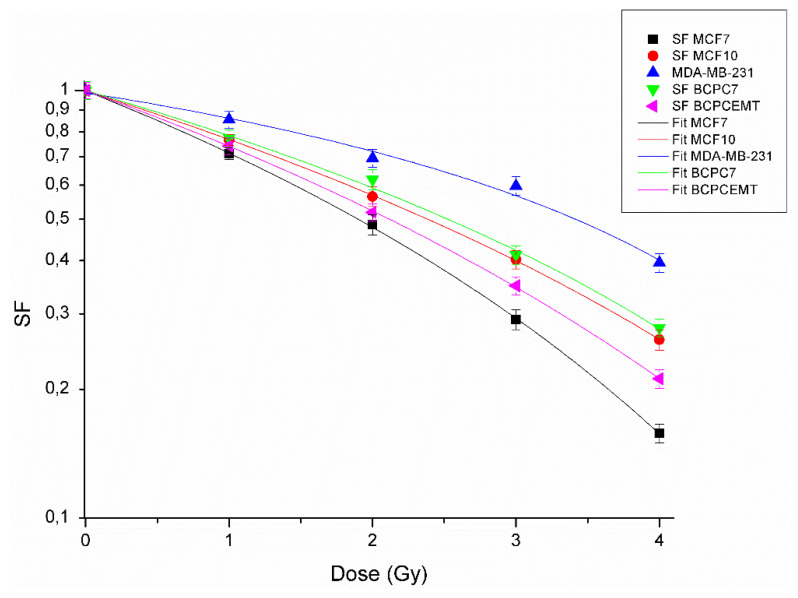
Clonogenic survival curves of breast cancer (BC) cells exposed to different doses of therapeutic X-rays. The data shown represent the mean values plus errors from three independent experiments.

In order to carry out a radiobiological characterization, the different BC cells were irradiated with increasing doses (1, 2, 3, and 4 Gy) of X-rays by a medical linear accelerator, as described in the Methods section. The surviving fraction (SF) values, calculated by clonogenic survival assay, are reported in the Supplementary Materials, Table S1. These values were plotted to obtain dose-response curves, according to the LQ model (Figure 1).

The dose–response curves show that the sensitivity to radiation treatments was different among the five BC cell populations; in particular, the MDA-MB-231 cells were the most radioresistant and the MCF7 cells the most radiosensitive cell lines.

In addition, we estimated the LQ parameters, α and β, for all BC cells, to give an insight of their intrinsic cell radiosensitivity. The α, β, and α/β parameters were calculated by fitting clonogenic survival data, and they are shown in Table 1.

### 3.2. Experimental LSR

**Figure 2:**
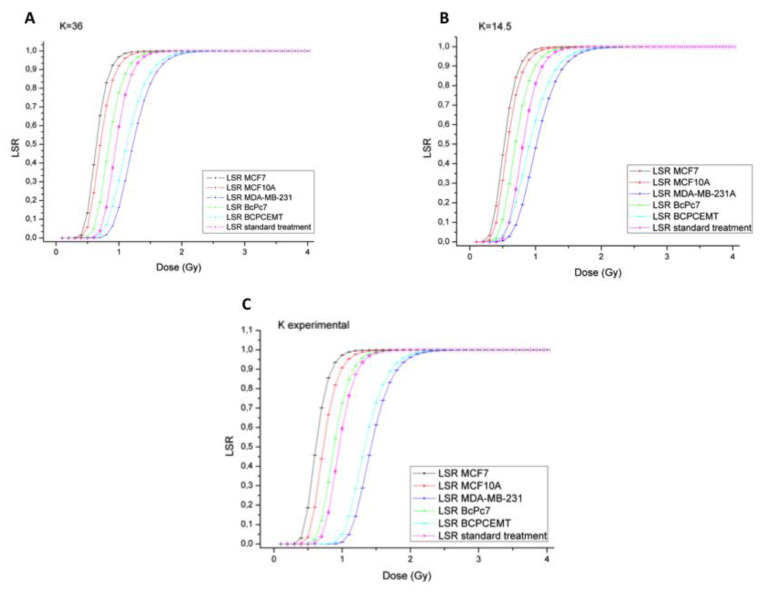
Local disease-free survival rate (LSR) curves with k = 36 (**A**) and k = 14.5 (**B**), as well as *k* values experimentally calculated (**C**) on BC cells exposed to increasing doses of therapeutic X-rays. The data shown are represented with their own error in *x* and *y*.

We calculated an experimental LSR using Equation (4), starting from the experimental α/β ratio, doubling time T_d_, and the *k* values for each cell culture. We also compared these experimental data with those obtained with the standard treatment of BC, where a theoretical α/β ratio equal to 3 is usually considered. LSR graphs that were obtained with *k* values reported in the literature, i.e., k = 36 [23,31] (Figure 2A) and k = 14.5 (Figure 2B), as well as those with *k* values experimentally measured (Figure 2C), are reported below.

An analysis of the above-reported graphs demonstrates that the dose required to reach an optimal LSR in triple-negative BC cells (both immortalized and primary), such as MDA-MB-231 and BcPcEMT, should be higher than the current 2 Gy for fraction for each *k* value. On the other hand, for the less aggressive cell line, MCF7, values lower than 2 Gy for fraction could be adopted. Moreover, for the primary tumor cell culture, BcPc7, and for the non-tumorigenic MCF10A cell line, it is sufficient to administer 2 Gy, at least for the experimental *k* and k = 36, in order to obtain an LSR(*D*) of 100%.

A summary of the k-dependent dose for the fraction for each cell type to obtain an LSR equal to 100% is reported in Table 2.

## 4. Discussion

Progress in the field of molecular diagnostics has led to a highlight of peculiar biological “fingerprints” from which the efficacy of a specific therapeutic plan depends. Especially for cancer therapy, both for chemotherapy and RT, clinicians should consider that the success of the treatment cannot be independent from the personal genetic assets of the patient to be treated [34]. It follows that a therapeutic approach generally based on the standard guidelines for a given neoplasm must necessarily imply a personalized path, also taking into account the biological features of patients. If on the one hand, the RT success could rely on patient features, then on the other hand, tumor heterogeneity represents a great challenge to overcome for their specific treatment. That is the case with regard to the different response to treatments observed in several BC subtypes [35], especially in the TNBCs [36].

RT effects on cancer cells have been evaluated widely for many years.

The LQ model was the first mathematical model developed to study the RT response treatment in 1966, and today it is still validly recognized. The Poisson tumor control probability (TCP) or LSR model is generally used in RT, and it refers to the probability of removing all cancer cells after RT treatment. The Poisson TCP formula follows a binomial distribution; however, when a statistical population is very large, as happens for a cancer cell population [37], it is more appropriate to apply a Gaussian distribution [38,39].

In our approach, we experimentally calculated both *α* and *β* values for BC cell cultures and for one non-tumorigenic breast cell line, to experimentally evaluate the LSR.

The basic principles of RT fractionation are useful to better understand the LSR (D) model validity we described. Indeed, the RT fractionation plan relies on the idea that cancer treatment by means of RT must guarantee the maximization of tumor cell eradication. This concept is clinically referred to as local tumor control [40]. On this basis, the aim of this work is to move towards the development of a more personalized therapeutic approach for BC. We do not speculate on the physical features and radiation beam conformity commonly used in clinical practice, but rather we propose the application of an LSR model that takes into account the cancer patient-specific biological parameters. The calculation of an experimental LSR model could represent a valuable tool for the further development of personalized RT treatments.

Our radiobiological approach was developed starting from the generalized quadratic linear model, and considering the local disease-free survival rate (LSR) for breast radiation treatment. This model is dependent on four variables, namely

- dose per fraction to achieve controlled death of cancer cells;
- the intrinsic radiosensitivity values α and β;
- *k*, which represents tumor clonogens;
- $T_{d}$ or the doubling time.

The novelty of our work is obtaining each parameter experimentally from each cell culture analyzed, unlike other previously published works, where these four parameters were minimized [29]. The *α*/*β* ratio, shown in Table 1, cannot act as a stand-alone feature that leads to the establishment of a new fractionation schedule. The reassessment in RT plans, in fact, could be dependent on the contribution of several factors that concurrently must be taken into consideration to obtain an optimal LSR. In order to show how the α/β ratio alone is not sufficient to describe the radiotherapy effectiveness, we analyzed the MCF7 and the MDA-MB-231 SF values, considering their *α*/*β* ratios of 6.47 and 3.79, respectively. Taking into account the fitting survival curves obtained by the analysis of the LQ model only (Figure 1), the MDA-MB-231 cell line needs an increase in dose of 77% in comparison to the MCF7, in order to achieve a cell killing of 50%. However, considering the dose calculated to obtain LSR = 100% with our method, the MDA-MB-231 cells need twice the dose administered to the MCF7; otherwise, there will be an increase of 100%.

Clinical success in oncology relies strictly on the peculiar biological asset of the tumor itself. However, the “one size fits all” theory still represents the most common approach, especially for radiation therapy. TNBCs are a typical example of the inconsistency of a unique therapeutic criteria for BC. In fact, due to their lack of specific biomarkers, targeted or endocrine therapies are ineffective and no exclusive strategies are feasible [11,41]. In addition, TNBCs display an increased radioresistance with respect to other breast tumor subtypes, and therefore it is extremely important to deliver a proper radiant dose for fraction that is adequate for the radiobiological cancer characteristics [42].

Several mathematical models are used to predict the response to radiotherapy. Some complex ones have been developed considering the total volume and irregular nature of the tumor mass, tumor reoxygenation capabilities, stage, and tumor grading. Nevertheless, each model is based on the tumor survival rate, which is calculated by means of the LQ model and directly depends on two radiobiological parameters that influence the tumor response, i.e., the *α*, the *β*, and their ratio (*α*/*β*). Radiation treatment schedules for all BC subtypes are currently and surprisingly administered using the unique and standard *α*/*β* ratio of 3 [43]; it follows that the success of the treatment is highly variable among affected patients. Moreover, it should be mentioned that conventional radiotherapy is characterized by the deposition of energy also within healthy organs close to the target, e.g., the lungs and heart in the case of breast tumors. On the other hand, radioresistant forms should be treated with a higher total dose with respect to the more radiosensitive ones. In such cases, to avoid the detrimental and intolerable effects on healthy tissues, other radiation therapy approaches, with a more precise deposition of the dose (like hadrontherapy), should be considered [44].

Our study suggests a mathematical model to better rationalize the therapeutic approach, which should be evaluated case by case, basing the approach on the specific biological traits of the tumor. From a short- and a long-term point of view, clinical success in oncology practice is achieved by the total eradication of the tumor, and the concurrent mitigation of side effects cannot be thoroughly avoided. Therefore, in terms of clinical significance, a modulation of the dose for fraction must be chosen in accordance to tumor radioresistance or radiosensitivity.

It should be underlined that our work has been based on an in vitro approach, and it should be considered as a seminal one. In the future, our data will be further implemented testing our thesis in an in vivo experimental model, in order to design more personalized RT protocols.

## 5. Conclusions

Heterogeneity is a cancer trademark, and it represents a main issue with regard to defining a “gold-standard” treatment for cancer care. In recent years, clinicians have been progressively moving towards the development of more personalized therapeutic regimens, with the support of new imaging and molecular diagnostic techniques. Here, we propose an alternative method for the calculation of LSR parameters on different BC cell cultures, representative of breast cancer heterogeneity. Our results could hopefully be useful for reconsidering the standardization of RT plans for BC, as the standard treatments are commonly based on a unique *α*/*β* ratio (3 Gy) and on a fixed dose of 2 Gy for fraction.

## Figures and Tables

**Table 1:** The α, β, and α/β values calculated by the linear quadratic (LQ) model application.

BC Cells	α (Gy^−1^)	β (Gy^−2^)	α/β (Gy)
MCF7	0.285 ± 0.012	0.044 ± 0.003	6.47 ± 0.52
MCF10A	0.236 ± 0.007	0.024 ± 0.002	9.83 ± 0.87
MDA-MB-231	0.110 ± 0.034	0.029 ± 0.010	3.79 ± 2.24
BcPc7	0.203 ± 0.022	0.029 ± 0.006	7.00 ± 1.63
BcPcEMT	0.264 ± 0.008	0.030 ± 0.002	8.83 ± 0.64

The different α/β ratio values obtained show cell response variability to radiation treatments. Therefore, their radiosensitivity may be affected as mentioned above, by their intrinsic molecular features.

**Table 2:** Dose values for fraction calculated to obtain an LSR(*D*) of 100% in the three conditions, analyzed with an experimental *k* (k exp), k = 36 and k = 14.5.

BC Cells	Dose (Gy) [k exp]	Dose (Gy) [k = 36]	Dose (Gy) [k = 14.5]
MCF7	1.5	1.5	1.5
MCF10A	2.0	2.0	2.0
MDA-MB-231	2.0	2.0	1.8
BcPc7	2.9	2.9	2.8
BcPcEMT	2.9	2.8	2.6

## Supplementary Materials

The following are available online at https://www.mdpi.com/2075-4426/10/4/177/s1, Table S1: The surviving fraction values (SF) of BC cell types after X ray irradiation with doses of 1, 2, 3 and 4 Gy; Table S2: Chi squared values (X^2^) and adjusted R^2^ values obtained by statistical analysis.
