# Supplementary material for: Local Disease-Free Survival Rate (LSR) Application to Personalize Radiation Therapy Treatments in Breast Cancer Models
Source: J Pers Med. 2020 Oct 17;10(4):177. doi: 10.3390/jpm10040177 (PMC7712665; doi:10.3390/jpm10040177)
Supplement: Supplementary file 1 [file jpm-10-00177-s001.pdf]

**Supplementary file 1**

**The surviving fraction values (SF) of BC cell types after X ray irradiation with doses of 1, 2, 3 and 4 Gy**

| Dose (Gy) | SF (MCF7) | SF (MCF10A) | SF (MDA-MB-231) | SF (BcPc7) | SF (BcPcEMT) |
|---|---|---|---|---|---|
| 0 | 1.000±0.045 | 1.000±0.046 | 1.000±0.048 | 1.000±0.049 | 1.000±0.044 |
| 1 | 0.711±0.021 | 0.766±0.022 | 0.854±0.038 | 0.773±0.035 | 0.741±0.033 |
| 2 | 0.485±0.026 | 0.565±0.030 | 0.694±0.033 | 0.619±0.034 | 0.519±0.024 |
| 3 | 0.291±0.016 | 0.402±0.020 | 0.598±0.030 | 0.412±0.021 | 0.349±0.017 |
| 4 | 0.158±0.008 | 0.261±0.015 | 0.396±0.020 | 0.278±0.014 | 0.212±0.011 |

**Supplementary file 2**

**Chi squared values ($\chi^2$) and adjusted $R^2$ values obtained by statistical analysis**

| BC cells | $\chi^2$ | Adjusted $R^2$ |
|---|---|---|
| MCF7 | 1.1 | 0,999 |
| MCF10A | 0.99 | 0,999 |
| MDA-MB-231 | 1.2 | 0,983 |
| BCPC7 | 1.4 | 0,996 |
| BCPCEMT | 1.2 | 0,999 |
